# Author Correction: The impact of sleep, physical activity and sedentary behaviour on symptoms of depression and anxiety before and during the COVID-19 pandemic in a sample of South African participants

**DOI:** 10.1038/s41598-022-08624-z

**Published:** 2022-03-15

**Authors:** R. Lewis, L. C. Roden, K. Scheuermaier, F. X. Gomez-Olive, D. E. Rae, S. Iacovides, A. Bentley, J. P. Davy, C. J. Christie, S. Zschernack, J. Roche, G. Lipinska

**Affiliations:** 1 UCT Sleep Sciences and Applied Cognitive Science and Experimental Neuropsychology Team (ACSENT), Department of Psychology, University of Cape Town, Cape Town, South Africa; 2 Department of Family Medicine, Faculty of Health Sciences, University of the Witwatersrand, Johannesburg, South Africa; 3 Department of Human Kinetics and Ergonomics, Rhodes University, Grahamstown, South Africa; 4 MRC/Wits Rural Public Health and Health Transitions Research Unit (Agincourt), School of Public Health, Faculty of Health Sciences, University of the Witwatersrand, Johannesburg, South Africa; 5 Brain Function Research Group, School of Physiology, Faculty of Health Sciences, University of the Witwatersrand, Johannesburg, South Africa; 6 Division of Exercise Science and Sports Medicine, Department of Human Biology, Faculty of Health Sciences, University of Cape Town, Cape Town, South Africa; 7 Faculty Research Centre for Sport, Exercise and Life Sciences, School of Life Sciences, Faculty of Health and Life Sciences, Coventry University, Coventry, CV1 2DS UK

Correction to: *Scientific Reports* https://doi.org/10.1038/s41598-021-02021-8, published online 15 December 2021

The Funding section in the original version of this Article was omitted. The Funding section now reads:

“G.L. is supported by the National Research Foundation (NRF; South Africa), Competitive Support for Unrated Research (CSUR) grant number 116229.”

The original Article has been corrected.

